# Association of GILZ with MUC2, TLR2, and TLR4 in Inflammatory Bowel Disease

**DOI:** 10.3390/ijms24032235

**Published:** 2023-01-23

**Authors:** Luigi Cari, Lucrezia Rosati, Giuseppe Leoncini, Eleonora Lusenti, Marco Gentili, Giuseppe Nocentini, Carlo Riccardi, Graziella Migliorati, Simona Ronchetti

**Affiliations:** 1 Pharmacology Division, Department of Medicine and Surgery, University of Perugia, P.le L. Severi 1, 06132 Perugia, Italy; 2 First Pathology Division, Department of Pathology and Laboratory Medicine, Fondazione IRCCS Istituto Nazionale dei Tumori, Via G. Venezian 1, 20133 Milano, Italy

**Keywords:** GILZ, IBD, mucins, TLR

## Abstract

Ulcerative colitis (UC) and Crohn’s Disease (CD) are chronic relapsing inflammatory diseases that are caused by genetic, environmental, and immune factors. Treatment strategies are currently based on symptomatic control by immunosuppression. The glucocorticoid-induced leucine zipper (GILZ), a mediator of several effects of glucocorticoids, was recently found to be secreted by goblet cells and play a role in inflammatory bowel disease (IBD). This study investigates which genes *GILZ* is associated with in its role in intestinal barrier functions. We examined datasets from the Gene Expression Omnibus (GEO) and ArrayExpress profiles of the gut of healthy subjects (HSs), as well as UC and CD patients. The human colonic epithelial HT29 cell line was used for in vitro validation experiments. *GILZ* was significantly correlated with *MUC2*, *TLR2*, and *TLR4*. In particular, an inverse correlation was found between the *GILZ* and *MUC2* in HS and patients with IBD, mostly in those with an active disease. Further, direct pairwise correlations for *GILZ*/*TLR2* and *GILZ*/*TLR4* were found in HSs and UC patients, but not in CD patients. Overall, our results reveal the crosstalk at the transcription level between the *GILZ*, *MUC2*, and *TLRs* in the mucosal barrier through common pathways, and they open up new perspectives in terms of mucosal healing in IBD patients.

## 1. Introduction

Inflammatory bowel disease (IBD), which includes ulcerative colitis (UC) and Crohn’s disease (CD), is characterized by chronic relapsing inflammation of the gastrointestinal (GI) tract that causes immune-mediated mucosal damage. The etiology of IBD has not been thoroughly elucidated so far, but it is thought to result from environmental, immune, and genetic factors [1]. Gut homeostasis is maintained through a complex interplay between the following functional compartments: (1) the luminal environment, including the microbiota; (2) the epithelial lining with the overlying mucus layer; and (3) the mucosa-associated immune system. Both the structural composition of the intestinal mucus barrier (IMB) and the intercellular junctions ensure optimal mucosal permeability and interaction between luminal microorganisms and immune effectors [2].

The IMB is composed of several types of gel-forming (GF) and transmembrane mucins (MUC) produced by goblet cells, as well as of other bioactive factors, e.g., trefoil factor 1–3, which are involved in the lumen–mucosa interplay [3]. MUC2 is a predominant GF mucin found throughout the gut; thus, any deficits in both MUC2 synthesis and secretion lead to IMB impairment, directly exposing the gut mucosa to the luminal content. This can promote bacterial endocytosis by goblet cells, binding of pathogen components to toll-like receptors (TLRs) 2, 1, 4, and 5, and a reactive increase in the luminal delivery of MUC2. However, protracted bacterial stimuli are known to induce neutrophil recruitment in the *lamina propria* and inhibit the secretion of MUC2 by goblet cells, thus leading to a decrease in the MUC2 levels and an increase in the vulnerability of the IMB during active mucosal inflammation [4]. This effect on the IMB triggers the host immune response against luminal microorganisms. This response involves the activation of TLR-expressing innate immune cells via ligand interaction, intracellular signaling, and consequent transcription of proinflammatory cytokines.

TLR2 and TLR4 are expressed in both enteroendocrine and goblet cells in the large bowel, whereas they are expressed at low levels in absorptive enterocytes and Paneth cells in the small intestine [5]. TLRs exert several functions in the gut, including regulation of permeability during infection [6], tight junction translocation [7,8], and MUC2 secretion [9]. Interestingly, TLR4 activation has been found to increase the proportion of goblet cells under both in vivo and in vitro conditions by promoting cellular differentiation towards goblet cell lineages [10,11].

There is growing evidence to suggest that the IMB is composed of MUCs and other bioactive compounds that are delivered to the intestinal environment. Recently, GILZ was identified as one of these compounds: it is expressed by epithelial cells in the ileum and colon, and its expression is driven by inflammatory reactions. In particular, GILZ is expressed both by goblet cells and enteroendocrine cells in the normal intestinal mucosa. During active disease, its expression is reduced or is absent in goblet cells, but it is restored during the quiescent period [12]. *GILZ* is an early glucocorticoid-induced gene that mediates several functions of glucocorticoids, which mainly include their anti-inflammatory effects [12,13,14]. GILZ has previously been found to be involved in IBD in mouse models of experimental colitis. In particular, *GILZ* ablation in Treg or B cells exacerbated DNBS-induced colitis in T-conditional [15] or B-conditional *GILZ*-KO mice, respectively [16]. Furthermore, granulocytes of *GILZ*-KO mice were found to be more activated than controls in the gut of DNBS-induced colitic mice, thus worsening the disease symptoms [17]. In a model of DSS-induced colitis, the treatment with a recombinant TAT-GILZ protein ameliorated the severity of the disease by improving the permeability and upregulating the expression of the tight junction protein zonula occludens-1 (ZO-1). This GILZ-mediated effect provided an optimal environment for the colonization of beneficial bacteria [18]. Therefore, GILZ exerts several functions on multiple cell types, all contributing to gut homeostasis. In the human gut, GILZ was found to be expressed in the goblet and enteroendocrine cells with a potential secretory role, and its expression was reduced or even absent in active disease in both UC and CD [12]. The identification of this novel role of GILZ in the gut luminal environment led us to hypothesize that GILZ could regulate or be associated with MUCs and other IMB components. The present work aims to study partner genes that correlate with *GILZ* dynamics in the mucosal barrier to affect its function in both healthy subjects (HSs) and those with IBD. To this end, publicly available data from whole human genome arrays deposited in the Gene Expression Omnibus (GEO) and ArrayExpress databases were analyzed, in both healthy and IBD tissues, which were divided into inflamed (active disease) and non-inflamed (quiescent disease) tissues [19,20]. The results indicated that *GILZ* shows significant correlations with *MUC2*, *TLR2*, and *TLR4*, and these findings were validated through in vitro experiments on human cell lines.

## 2. Results

### 2.1. GILZ Expression in GI Tract Tissue from Healthy and IBD Patients

**Figure 1:**
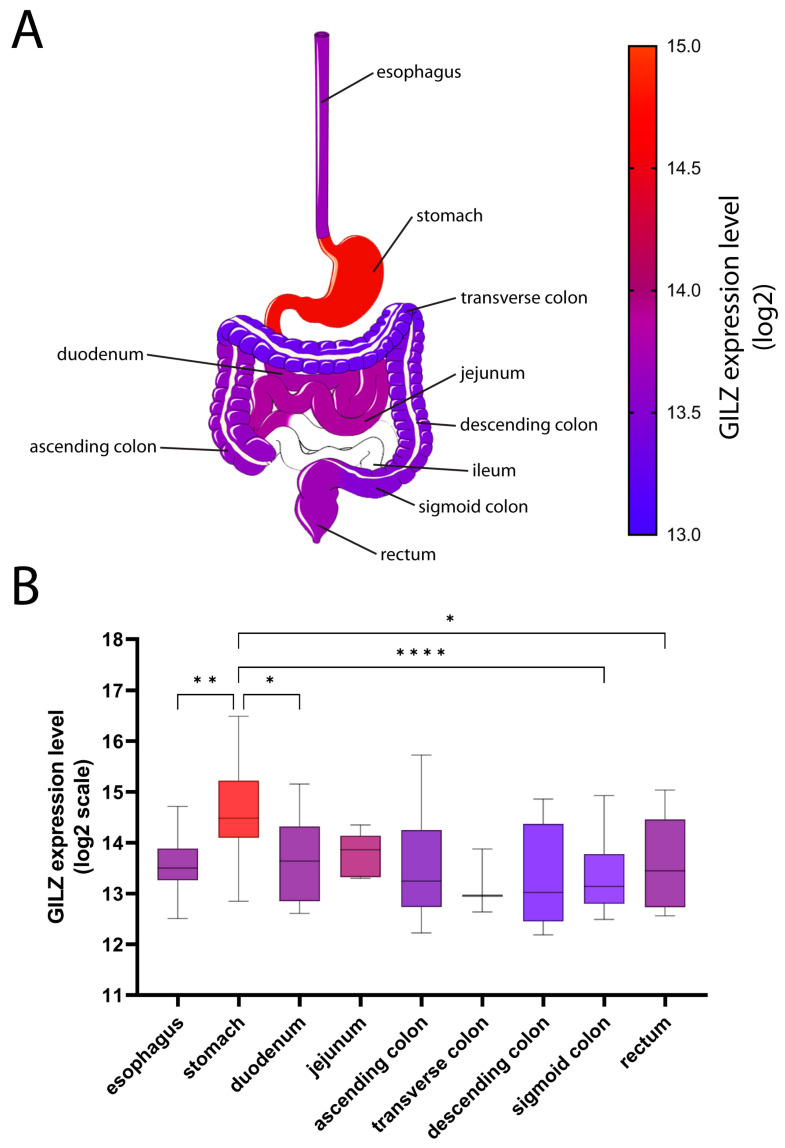
GILZ expression in different parts of the gut. (**A**) GILZ expression levels along the GI tract range from 13 (blue) to 15 (red) on the log2 scale. (**B**) Mean expression levels of GILZ are shown as a box-and-whiskers plot (Tukey). The scale for the box colors is the same as that for (**A**). *p*-values < 0.05 were considered statistically significant; *p* < 0.05 = *; *p* < 0.01 = **; *p* < 0.0001 = ****.

GILZ protein is expressed in intestinal goblet and enteroendocrine cells, as well as in the gastric mucosa, but it is not expressed in gastric chief cells and intestinal Paneth cells [12]. To thoroughly explore the GI tract, we extracted and analyzed 284 samples from healthy subjects obtained along the GI tract with the help of the Genevestigator V3 suite. *GILZ* mRNA was found to be expressed at high levels in the stomach (14.62 ± 0.85 log2), with significant differences versus the esophagus (13.60 ± 0.68 log2), duodenum (13.72 ± 0.87 log2), sigmoid colon (13.37 ± 0.78 log2), and rectum (13.62 ± 0.87 log2) (Figure 1). Ileal specimens were not available in the database, but we have previously demonstrated *GILZ* expression in the ileum [12].

**Figure 2:**
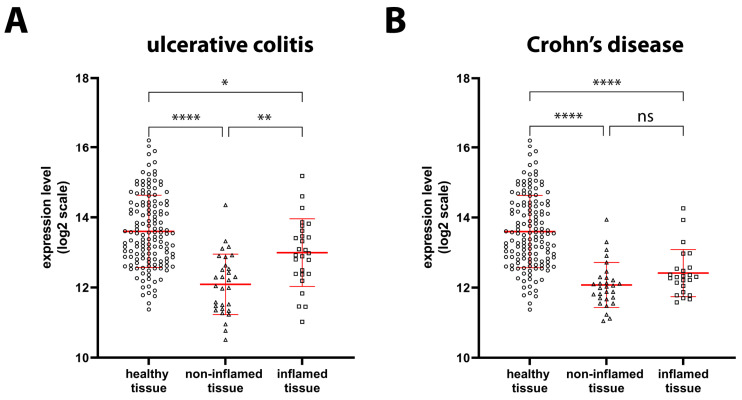
*GILZ* in healthy, UC, and CD tissues. (**A**) *GILZ* gene expression levels based on microarray data were compared between healthy tissue (circles, *n* = 142), as well as non-inflamed (triangles, *n* = 30) and inflamed (squares, *n* = 27) tissue from UC patients. (**B**) *GILZ* gene expression levels based on microarray data were compared between healthy tissue (circles, *n* = 142), as well as non-inflamed (triangles, *n* = 29) and inflamed (squares, *n* = 24) tissue from CD patients. Data are shown by using a scatter plot, with each dot representing a single patient; values are shown in log2 scale and expressed as mean ± SD. *p*-values <0.05 were considered statistically significant; *p* < 0.05 = *; *p* < 0.01 = **; *p* < 0.0001 = ****. ns = non-significant.

We have previously obtained data about the reduced protein expression of GILZ during the active phase of UC and CD and confirmed the corresponding reduction in mRNA expression in a fairly large number of patients [12]. To confirm our previous observations, we analyzed *GILZ* expression data from the GEO and ArrayExpress databases. Our findings indicated a significant reduction in the expression of *GILZ* in UC mucosal biopsy samples of both inflamed (−35%) and non-inflamed (−65%) tissues compared to samples of HSs (Figure 2A). A similar significant reduction in *GILZ* expression was observed in CD patients, in both inflamed (−56%) and non-inflamed (−65%) tissues (Figure 2B). Thus, our previous results were confirmed by the present ones.

### 2.2. Expression of Genes Involved in Intestinal Barrier Function in Healthy and IBD Patients

**Figure 3:**
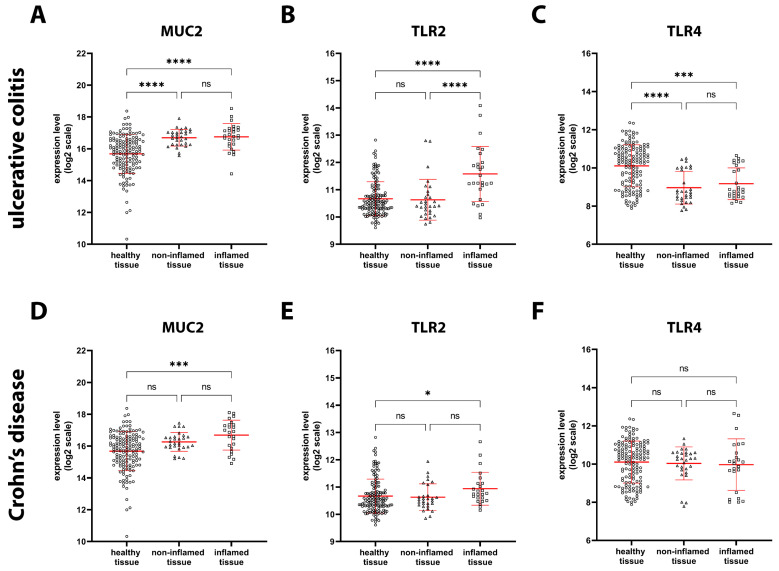
*MUC2*, *TLR2*, and *TLR4* expression in healthy, UC, and CD tissue. *MUC2* (**A**), *TLR2* (**B**), and *TLR4* (**C**) gene expression levels based on microarray data were compared among healthy tissue (circles, *n* = 142), as well as non-inflamed (triangles, *n* = 30) and inflamed (squares, *n* = 27) tissue from UC patients. *MUC2* (**D**), *TLR2* (**E**), and *TLR4* (**F**) gene expression levels based on microarray data were compared between healthy tissue (circles, *n* = 142), as well as noninflamed (triangles, *n* = 29) and inflamed (squares, *n* = 24) tissue from CD patients. Data are shown by using a scatter plot, with each dot representing a single patient; values are shown in log2 scale and expressed as mean ± SD. *p*-values < 0.05 were considered statistically significant; *p* < 0.05 = *; *p* < 0.001 = ***; *p* < 0.0001 = ****. ns = non-significant.

Next, we analyzed the expression of *MUC2*, *TLR2*, and *TLR4*, which are involved in several functions, including mucosal barrier maintenance and pathogen recognition. MUC2 is the most abundant gel-forming mucin in the gut and is secreted by goblet cells, and its function is to protect the inner mucus layer from bacteria trapped in the outer layer. Our analysis showed that *MUC2* mRNA expression was significantly increased in both inflamed (+110%) and non-inflamed (+100%) tissue specimens from UC patients (Figure 3A). Conversely, it was significantly increased only in inflamed (+100%) specimens from CD patients (Figure 3D).

TLRs are membrane receptors that recognize so-called “pathogen-associated molecular patterns”, or PAMPs. *TLR2* was significantly upregulated in inflamed UC (+88%) and CD (+21%) tissue, whereas it remained unchanged in noninflamed tissues compared to healthy tissue (Figure 3B,E). Since all cells in the biopsy samples were analyzed, infiltrated granulocytes could have contributed to the increase in *TLR2* expression in inflamed tissues. Interestingly, *TLR4* expression was significantly decreased in both inflamed (−50%) and non-inflamed (−55%) UC tissue (Figure 3C), whereas it was unchanged in inflamed and non-inflamed CD tissue (Figure 3F).

### 2.3. Correlation of GILZ Expression with MUC2, TLR2, and TLR4 Expression

**Figure 4:**
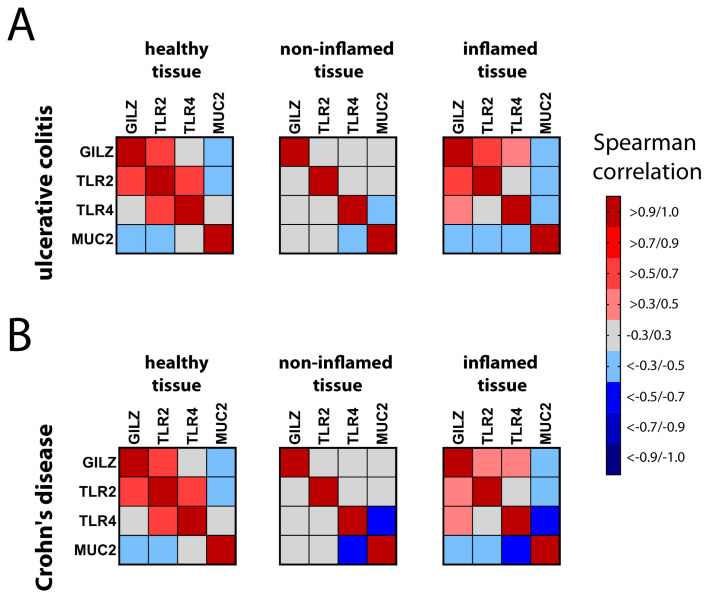
Heat map showing pairwise comparisons of *GILZ*, *MUC2*, *TLR2*, and *TLR4* expression. Pairwise comparisons of the genes were made using Spearman’s correlation analysis for healthy control tissue (in (**A**,**B**)) and inflamed/non-inflamed UC (**A**) and CD (**B**) tissues. Positive correlations are presented in the upper red part of the scale, while negative correlations are presented in the lower blue part of the scale. Correlation values between 0.3 and −0.3 were not considered significant and are shown in grey.

To examine whether expression of the *GILZ* gene is associated with expression of the *MUC2*, *TLR2*, and *TLR4* genes, we performed a correlation analysis on selected pairs of the four genes. We generated a heat map for healthy controls, UC patients, and CD patients, in order to compare inflamed and non-inflamed tissues from both UC (Figure 4A) and CD (Figure 4B) patients. We considered only positive correlation values (r > 0.3, red) and negative correlation values (r < 0.3, blue) to be biologically significant.

**Figure 5:**
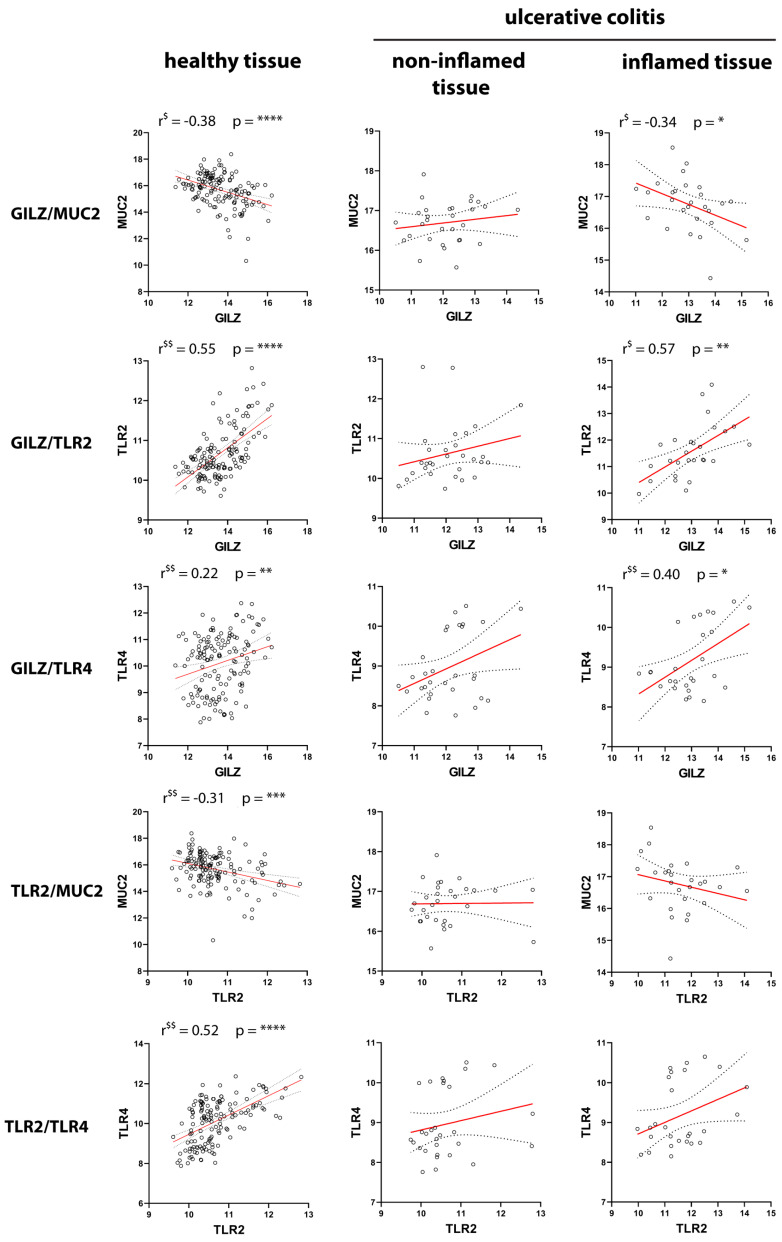

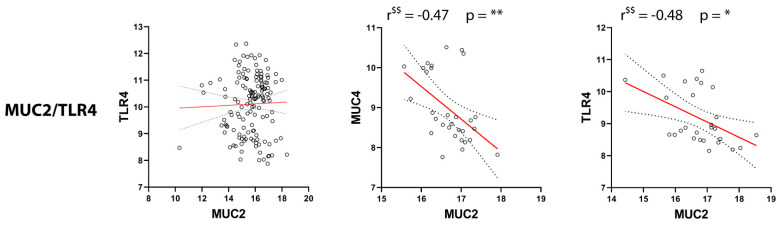
Pairwise correlations between *GILZ*, *MUC2*, *TLR2*, and *TLR4* in healthy and UC tissues. Correlations between the expressions of the four genes in healthy control tissue (**left** panels), noninflamed tissue (**middle** panels), and inflamed tissue (**right** panels) from UC patients are shown. $: significant according to Pearson’s correlation analysis; $$: significant according to Spearman’s correlation analysis. *p*-values < 0.05 were considered statistically significant; *p* < 0.05 = *; *p* < 0.01 = **; *p* < 0.001 = ***; *p* < 0.0001 = ****. Non-significant correlations (*p* > 0.05) were not reported.

**Figure 6:**
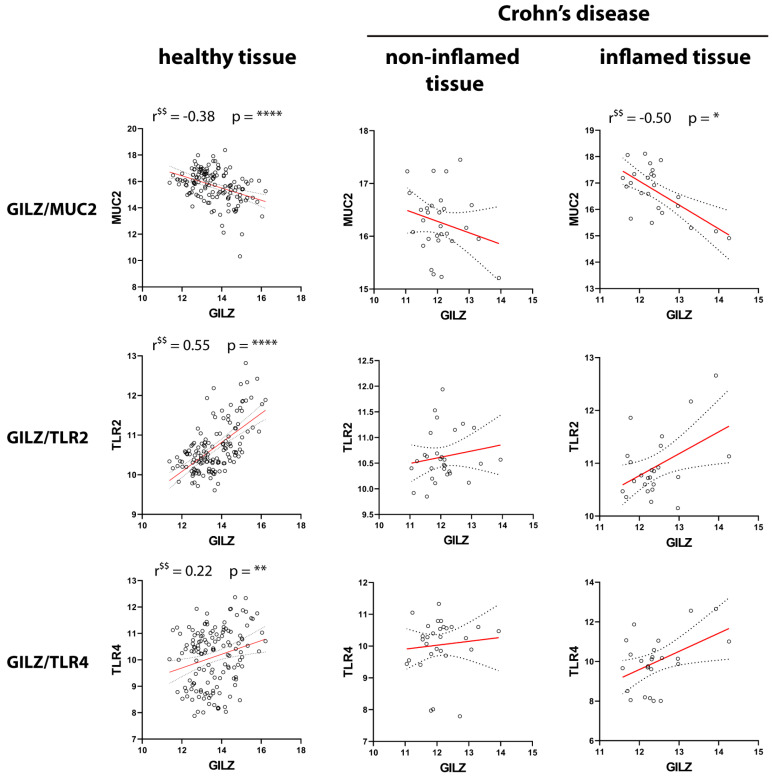

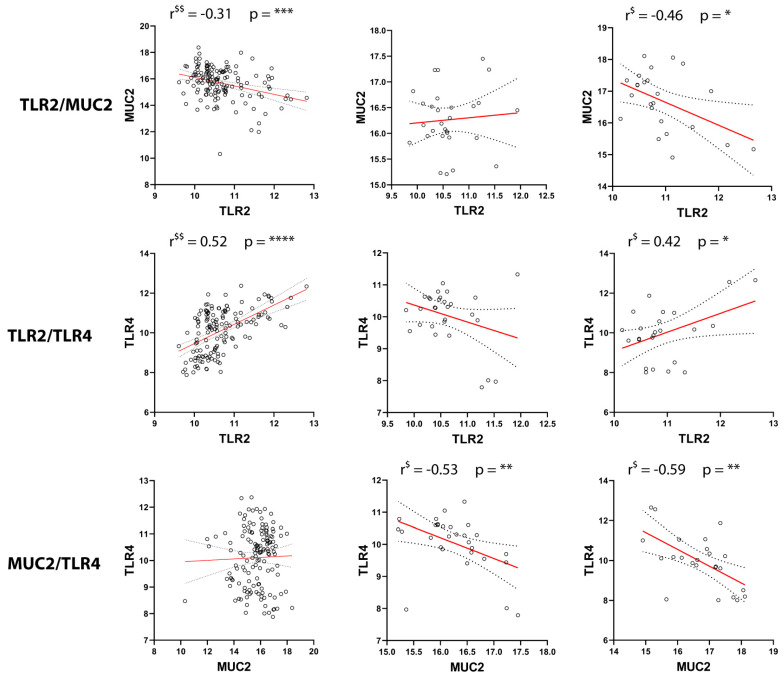
Pairwise correlations between *GILZ*, *MUC2*, *TLR2*, and *TLR4* in healthy and CD tissues. Correlations between the expressions of the four genes in healthy control tissue (**left** panels), noninflamed tissue (**middle** panels), and inflamed tissues (**right** panels) from CD patients are shown. $: significant according to Pearson’s correlation analysis; $$: significant according to Spearman’s correlation analysis. *p*-values < 0.05 were considered statistically significant; *p* < 0.05 = *; *p* < 0.01 = **; *p* < 0.001 = ***; *p* < 0.0001 = ****. Non-significant correlations (*p* > 0.05) were not reported.

The data shown in Figure 4 are presented in more detail in Figure 5 and Figure 6. In particular, Figure 5 presents the selected genes and their reciprocal correlations in UC tissue. The most interesting correlation was that between *GILZ* and *MUC2*, since both proteins are expressed in goblet cells. The negative correlation between *GILZ* and *MUC2* observed in healthy and inflamed tissue was lost in non-inflamed tissue, so the biological function of this interaction remains to be investigated. In contrast, *GILZ* was positively correlated with both *TLR2* and *TLR4* in inflamed tissues, but not in non-inflamed tissue. Further, in healthy controls, *GILZ* was correlated with *TLR2*, but not with *TLR4*. Interesting reciprocal correlations were observed between other gene pairs, too. For example, the positive correlation between TLR2 and TLR4 observed in healthy tissue was lost in both inflamed and non-inflamed tissue. With regard to the correlations between *TLRs* and *MUC2*, a positive physiological correlation was found between *TLR2* and *MUC2* in healthy tissue. However, this association was completely lost in inflamed and non-inflamed tissue. Interestingly, a negative correlation between *TLR4* and *MUC2* was observed only in both active and quiescent disease states.

Figure 6 shows the selected genes and their reciprocal correlations in CD tissue. To facilitate the comparisons, the healthy samples were the same as those in Figure 5. Similar to the observations for UC tissue, *GILZ* expression was negatively correlated with *MUC2* expression in inflamed CD tissues. In contrast to the findings for UC, the correlation of *GILZ* with *TLR2* was lost in disease tissue in both the active and quiescent disease states. Further, unlike the observations for UC, *TLR2* was negatively correlated with *MUC2* but positively correlated with *TLR4* in inflamed tissue. Finally, as observed for UC, *MUC2* expression was negatively correlated with *TLR4* in both inflamed and non-inflamed tissues. All the other reciprocal correlations were non-significant, which was indicative of independent gene regulation.

### 2.4. Effect of LPS and Proinflammatory Cytokines on the Expression of Genes Involved in Gut Barrier Function

**Figure 7:**
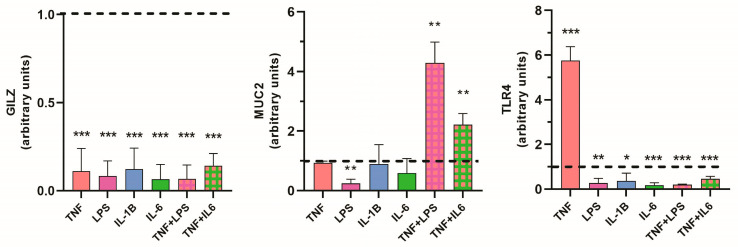
Quantitative PCR validation of bioinformatics data. HT29 cell extracts were used to measure *GILZ*, *MUC2*, and *TLR4* expression. Cells were treated with the indicated stimuli for 3 h. The expression of the genes was compared between treated and untreated (dotted line) control cells with the 2^−ΔCt^ method. Values are expressed as the mean ± SD for three different experiments; *p* < 0.05 = *; *p* < 0.01 = **; *p* < 0.001 = ***; *p* < 0.0001 = ****.

We used the HT29 cell line, which is a goblet-like cell line, to analyze changes in the expression of the selected genes in response to inflammatory stimuli that mimic the human pathology of IBD. First, we treated the HT29 cell line with proinflammatory cytokines or LPS for 3 h. Some groups received a combination of two stimuli. We observed significant downregulation of *GILZ* under each of the inflammatory conditions (Figure 7A). This confirmed the data from the bioinformatics analysis and those previously obtained from biopsy analyses [12]. *MUC2* expression was not affected by cytokines when the cells were treated with a single stimulus, but it was significantly upregulated when the stimuli were used in combination, that is, with both TNFα + LPS and TNFα + IL-6. These results are also similar to those obtained from bioinformatics analysis (Figure 7B). Interestingly, LPS, which binds to TLR4, significantly reduced *MUC2* mRNA expression when used alone. This finding is in line with the inverse correlation between *MUC2* and *TLR4* expression observed in UC and CD tissue, for both active and inactive disease (Figure 5 and Figure 6). This effect seems to be independent of the inflammatory state, as the addition of TNFα to LPS reversed this effect.

We studied *GILZ* regulation by proinflammatory cytokines in the Caco2 cell line, an epithelial intestinal cell line, but *GILZ* expression did not differ across the treated and untreated groups. This is consistent with the very low expression of *GILZ* in epithelial cells [12]. Unfortunately, we were not able to amplify *TLR2* either in HT29 or in Caco2 cells by the TaqMan or SYBR green techniques.

*TLR4* was found to be significantly downregulated in response to all the inflammatory stimuli, except for TNFα, in response to which its expression was highly increased. For all other proinflammatory stimuli, the changes observed in *TLR4* expression were similar to those observed for *GILZ* expression (Figure 7C) and were consistent with the positive correlation observed in inflamed tissue from UC patients (Figure 5).

## 3. Discussion

Gene microarray technology has been extensively used in the past few years to study the molecular basis of several diseases, including IBD. The focus of our study was *GILZ*-related genes and their mutual involvement in mucus barrier regulation in IBD. Both UC and CD are characterized by architectural effacement, mucin depletion, dense lymphocytic mucosal infiltration with basal plasma cells, and intermingled eosinophils. In the active disease, neutrophil infiltration and epithelial injuries, including erosion and ulceration across the superficial epithelium, as well as cryptitis and crypt abscesses, are characteristic. Nonetheless, UC displays a mucosal-restricted involvement limited to the colorectum, with a continuous pattern of mucosal involvement. Conversely, CD can involve the whole GI tract, with the terminal ileum being the most common site of onset. It displays a transmural pattern, thus promoting fissurations and strictures as major complications in long-standing disease, and skip lesions are commonly found. From a histological perspective, the epithelial injury is a predominant feature in the active UC, thus producing reactive glandular changes such as mucin depletion, which is less common in CD [1].

The active phase of the disease was recently found to be associated with GILZ down-regulation in goblet cells in both UC and CD, with GILZ dynamics being mostly related to neutrophil mucosal infiltration, as confirmed by the GILZ restoration during quiescence. Present data confirmed that the GILZ is expressed along the GI tract, with the highest expression level being found in the stomach. Wounding and healing represent the characteristic features of IBD. Such events deeply impact both the gut epithelial lining and the mucus barrier, which are regulated by dedicated molecular pathways and intricate cross-talks. Mucosal injury results from the derangement of such a homeostatic network due to pathogens or immune dysregulation, triggering acute inflammation [21].

MUC2 represents one of the major components of the mucus barrier, preventing the contact between luminal microorganisms and intestinal epithelial cells [4]. When barrier integrity is compromised, gut microbes and related products gain the access to the mucosa, triggering inflammation [22]. Intestinal epithelial cells (IECs) express the TLRs, among other pattern recognition receptors (PRRs), in order to counteract microbial invasion [21,23].

Our results revealed that the expression of *MUC2*, *TLR2*, and *TLR4* was affected, along with the expression of *GILZ*, in biopsy tissue samples from UC and CD patients. Particularly, MUC2 modulation has been previously studied in IBD, and there is a general agreement about the increase in *MUC2* mRNA expression in IBD [24,25,26]. However, some studies have reported a reduction in mucin synthesis based on protein immunolabeling experiments. Post-transcriptional abnormalities may be partly responsible for the discrepancy between the studies, as misfolded proteins are known to accumulate in the endoplasmic reticulum and cause a decrease in the amount of detectable MUC2 in the secretive granules [4,27,28]. Our evaluations show that *MUC2* expression levels were significantly increased in IBD tissue samples, as reported in several other studies. Interestingly, we found high *MUC2* levels in both active (inflamed) and quiescent (non-inflamed) UC, while high *MUC2* levels were found in active CD, but not in quiescent CD. These findings indicate different *MUC2* expression dynamics in UC versus CD. However, the high level of *MUC2* expression in inflamed tissues of both CD and UC may reflect an attempt to start a reparative process in the injured mucosal barrier.

An interesting finding in this study is the inverse correlation between *GILZ* and *MUC2* that was observed in healthy control tissue and in inflamed UC and CD samples. This inverse association was confirmed by our experiments on the HT29 cell line in which proinflammatory stimuli were found to upregulate *MUC2* expression and downregulate *GILZ* expression. As the inverse association between *GILZ* and *MUC2* observed in healthy tissue was retained in inflamed tissues and found in both UC and CD tissues, this finding may indicate a mutual gene regulation, regardless of the inflammatory status. This regulation seems to be lost in remitting IBD though. Accordingly, *MUC2*/*GILZ* co-expression has previously been observed in goblet cells and supports the inverse correlation observed here, as *MUC2* was expressed whilst *GILZ* expression was downregulated in mucosal samples of active IBD [12]. Taken together, our data suggest that GILZ may exert a regulatory role on MUC2 by hindering its excessive secretion in the gut and preventing an imbalance in the composition of the IMB. TLRs expressed on IECs play a role in microbial component recognition and triggering the immune response [29]. In health, intestinal TLR2 is expressed on the basolateral surface of the IECs lining the gut mucosa as well as in a subset of mononuclear cells that are predominantly located in the colonic *lamina propria*. TLR2 expression has been found to play a critical role in improving barrier function during injury and in tight junction preservation. However, in healthy tissue, it is expressed at low levels, and it does not seem to play a role in the maintenance of barrier function [30]. We found a positive correlation between *GILZ* and *TLR2* expression in healthy and UC tissues, but not in CD tissues. This suggests that GILZ may play an indirect role in modulating pathogen recognition, as supported by negative regulation of *TLR2* gene transcription by dexamethasone-induced GILZ in mouse neutrophils [31]. In addition, GILZ and TLR2 may play a role in common barrier functions, since TLR2 activation has been reported to elicit protective effects on tight junctions in animal models of colitis [32], and exogenously administered GILZ protein reduced intestinal permeability and promoted ZO-1 expression in a dextran sodium sulfate (DSS)-colitis model [18].

TLR4 is predominantly expressed in the colonic mucosa, specifically on its basolateral surface, but it is absent in the small intestine [29]. Previous studies have shown that interferon-γ and TNFα induce *TLR4* transcription, which in turn is required for the induction of cyclooxygenase 2 expression in DSS-induced colitis in mice [33,34]. Our results showed that *TLR4* expression was downregulated in UC tissue, supporting the pivotal role played by *TLR4* in triggering the immune response after bacterial stimulation [35]. No variation in *TLR4* expression was found in the data analysis on CD patients, even though some authors have reported that TLR4 is expressed on the apical membrane in CD mucosal samples [30]. *GILZ* and *TLR4* expression showed a positive correlation in inflamed UC tissues, but not in inflamed CD tissues, and this was confirmed by our in vitro results. Both sets of findings demonstrate that the expression of these two genes is significantly downregulated under inflammatory conditions. As observed for *MUC2*, *TLR4* expression was also different for UC and CD. The regulation patterns of these genes may reflect an attempt to restore the function of the intestinal epithelial barrier [36]. The inverse correlation between *MUC2* and *TLR4* in IBD is consistent with the findings of previous studies on the IEC-6 enterocyte cell line which showed that TLR4 silencing promoted MUC2 synthesis. In contrast, TLR4 overexpression in Caco2 cells resulted in the loss of MUC2 expression [37]. Yet, the administration of melatonin was found to promote the induction of MUC2-secreting goblet cells via TLR4 stimulation in a DSS-induced model of colitis. These findings are confirmed by other results from human cell lines [38].

The present study is limited by the absence of clinical correlations within a selected case series. Nonetheless, each result has been validated with in vitro experiments on the HT29 cell line under inflammatory stimuli. Another limitation is that we did not have the possibility to analyze human biopsies to explore the reciprocal correlation of these genes.

## 4. Materials and Methods

### 4.1. Datasets

Expression data from the Gene Expression Omnibus (GEO) database of whole human genome arrays [19] and the ArrayExpress Archive of Functional Genomics Data (ArrayExpress) [20], generated using the Affymetrix Human Genome-U133-Plus-2.0 platform, were downloaded and processed through the Genevestigator V3 suite (NEBION AG, Zurich, Switzerland) [39]. The microarray data in Genevestigator were normalized at two levels: robust multiarray average within experiments (through the Bioconductor package “affy” and a customized version of the package “affyExtensions”) and trimmed mean adjustment to a target for normalization between datasets. With regard to the latter, the trimmed mean is determined by calculating the mean of all the expression values in an experiment (across all samples) after excluding the top 5% and the bottom 5%. The combination of the two levels of normalization makes the data highly comparable across different experiments, thus making it possible to pool data without further normalization. The Genevestigator database was queried in October 2022. In addition to the inclusion and exclusion criteria reported in previous publications by the authors of the datasets [40,41,42,43,44], we included in the analysis only the arrays for mRNA samples that (1) were not obtained by laser capture microdissection of single cells and (2) were not subjected to in vitro experimental treatments. We extracted and considered data from 536 arrays of healthy and diseased tissue. Data were obtained on *GILZ* expression along the GI tracts of HSs (*n* = 284 from datasets GSE3526, GSE7307, GSE18105, GSE23878, GSE10714, GSE10191, GSE9686, GSE38713, GSE8671, GSE13911, GSE20916, GSE19826, GSE4183, GSE28177, E-MEXP-1828, E-MEXP-1823, GSE26886, and GSE43346), in the gut of UC (non-inflamed tissue: *n* = 30 from datasets GSE13367, GSE11831, GSE9452, E-TABM-118, and E-MEXP-2083; inflamed tissue: *n* = 27, from datasets GSE13367, GSE11831, GSE9452, E-TABM-118, and E-MEXP-2083) and CD patients (non-inflamed tissue: *n* = 29 from datasets GSE11831, GSE9452, E-TABM-118, and E-MEXP-1225; inflamed tissue: *n* = 24, from datasets GSE11831, GSE9452, E-TABM-118, E-MEXP-2083, and E-MEXP-1225), and in the colon/rectum tissue of HSs (*n* = 142 from datasets GSE7307, GSE8671, GSE18105, GSE20916, GSE23878, GSE4183, GSE10714, GSE10191, GSE10616, GSE9686, GSE38713, GSE43346, and GSE52746).

### 4.2. Gene Expression Analysis

Normalized gene expression data (expressed as log2 values) were downloaded from the Genevestigator V3 suite (NEBION AG, Zurich, Switzerland). *GILZ* expression was analyzed together with *MUC2*, *TLR2*, and *TLR4* expression since these genes were found to be involved in barrier function and microorganism recognition. *GILZ* expression was analyzed in the GI tract and gut mucosa samples from HSs and in biopsy tissue samples from UC and CD patients. *MUC2*, *TLR2*, and *TLR4* expression was analyzed in healthy mucosal gut samples and biopsy samples of UC and CD patients.

### 4.3. Cell Lines and In Vitro Treatments

The human colonic epithelial cell line HT29 was kindly provided by Dr. Efisio Puxeddu. HT29 cells were cultured in Dulbecco’s modified Eagle medium containing 10% fetal bovine serum, 1% nonessential amino acids, 1% sodium pyruvate, 50 IU/mL of penicillin, and 50 μg/mL of streptomycin at 37 °C in an atmosphere containing 5% CO_2_. Cells were seeded in 75 cm^2^ flasks and used at passage 10–12. At 18 h prior to exposure to the selected stimuli, cells were plated in 24-well plates (5 × 10^5^ cells/mL) containing the culture medium and incubated for 12 h at 37 °C in an atmosphere containing 5% CO_2_.

For validation studies, three separate experiments were conducted in which cells were treated with the following stimuli (at a dose of 10 ng/mL) for 3 h: recombinant human tumor necrosis factor α (TNFα), interleukin 1β (IL-1β), and interleukin 6 (IL-6) (all three purchased from Cell Guidance System, Cambridge, UK). Lipopolysaccharide (LPS) was purchased from Sigma-Aldrich (St. Louis, MI, USA). To strengthen the inflammatory milieu, specific combinations of TNFα + LPS and TNFα + IL-6 were also added. Stimuli, single or combined, were added in three wells per group, with a final concentration of 10 ng/mL for 3 h. This concentration was chosen within a range of concentrations used for in vitro studies with HT29 cell lines (5–100 ng/mL) [45]. A three-hour time of incubation was chosen since *GILZ* is an early gene, whose expression peaks at 3 h after induction [17,46]. Untreated cells were used as the control group. After 3 h, each well was washed with 800 µL of sterile PBS. 

### 4.4. RNA Extraction and Reverse Transcription

mRNA was extracted with the RNeasy Plus Micro Kit (Qiagen, Hilden, Germany), following the protocol of the manufacturer. Briefly, RLT Plus buffer was added to each well for direct lysis. After resuspension, the lysate was homogenized and transferred in a specific spin column. An amount of 50 ng of the extracted mRNA was converted to cDNA with the QuantiTect Reverse Transcription Kit (Qiagen, Hilden, Germany), and the cDNA was diluted before quantitative PCR by adding 40 µL of RNase-free water to reach a final volume of 60 µL of cDNA per sample.

### 4.5. Quantitative PCR

Quantitative PCR was performed in triplicate on the QuantStudio 1 Real-Time PCR System (Applied Biosystem, Waltham, MA, USA). The TaqMan^®^ gene expression assay protocol was used to detect the *GILZ* with a FAM™ probe (Hs00929365_m1; Thermo Fisher Scientific, Waltham, MA, USA). Eukaryotic 18 S rRNA was used as the endogenous control and detected with the VIC™ probe (Hs03003631_g1; Thermo Fisher Scientific) using TaqMan™ Gene Expression Master Mix (Applied Biosystems, Thermo Fisher Scientific, USA). *MUC2* and *TLR4* were quantified with the SYBR™ Green Assay protocol using SYBR™ Select Master Mix (Applied Biosystems, Thermo Fisher Scientific, USA), with the eukaryotic GAPDH gene as the endogenous control. The primers used were as follows in Table 1 (5′ to 3′):

Delta threshold cycles (ΔCt) were determined based on the difference between the target gene CTs and endogenous control CTs, and mRNA expression was evaluated using the CT cycle (2^−ΔCt^) method.

### 4.6. Statistical Analysis

Statistical analysis was conducted using Prism v.9.4.1 (GraphPad, San Diego, CA, USA). The Kolmogorov–Smirnov normality test was performed to analyze the distribution of data. *p*-values were calculated using the ordinary one-way ANOVA (Tukey) test for normally distributed data and the Kruskal–Wallis (Dunn) test for data with skewed distribution. To assess the correlations between gene expression levels, either Pearson’s correlation (for normally distributed data) or Spearman’s correlation (for non-normally distributed data) analysis was used. The heat map (correlation matrix) was generated by the software, accordingly, with the correlation coefficient (r) values. Only correlations with an r value of >0.3 or <−0.3 were considered to be biologically significant. For evaluation of the quantitative PCR results, an unpaired *t*-test was used to analyze the difference between the treatment and nontreatment (control) groups in the in vitro experiments. *P*-values < 0.05 were considered statistically significant; *p* < 0.05 = *; *p* < 0.01 = **; *p* < 0.001 = ***; *p* < 0.0001 = ****.

## 5. Conclusions

The present study represents the first attempt to investigate the crosstalk between GILZ and the molecular partners involved in barrier functions. Overall, *GILZ* correlated with *MUC2*, *TLR2*, and *TLR4* expression, as confirmed by in vitro validation studies on human cell lines. Our results contribute to identifying unrevealed functions exerted by GILZ as a critical regulator of barrier functions, being the intimate interplay linking GILZ, MUC2, and TLR2/4—a crucial factor involved in the gut homeostasis. Further studies are needed to translate such findings into future pharmacological perspectives.

## Figures and Tables

**Table 1:** The primers used for Quantitative PCR of MUC2 and TLR4.

Gene	Forward	Reverse
*MUC2*	ACTCTCCACACCCAGCATCATC	GTGTCTCCGTATGTGCCGTTGT
*TLR4*	CTGCCACATGTCAGGCCTTAT	AATGCCCACCTGGAAGACTCT
*GAPDH*	GCTCCTCCTGTTCGACAGTCA	GCAACAATATCCACTTTACCAG

## Data Availability

Not applicable.
